# IgG4-Related Autoimmune Pancreatitis Mimicking Pancreatic Cancer: A Report of Two Cases

**DOI:** 10.7759/cureus.65289

**Published:** 2024-07-24

**Authors:** Fatema Mohamed, Balasubramaniam Vijayan, Lokesh Saraswat

**Affiliations:** 1 Gastroenterology and Hepatology, Aberdeen Royal Infirmary Hospital, Aberdeen, GBR; 2 Radiology, Aberdeen Royal Infirmary Hospital, Aberdeen, GBR

**Keywords:** igg-4-related disease, igg4-related autoimmune pancreatitis, autoimmune pancreatitis type 1, pancreatic mass, igg4 disease

## Abstract

IgG4-related autoimmune pancreatitis (AIP) is a rare inflammatory condition characterized by elevated IgG4-positive plasma cells and lymphoplasmacytic infiltration in the pancreas. This disease responds to steroid therapy but can be challenging to differentiate from pancreatic cancer. In this paper, we present two cases of IgG4-related AIP presenting as pancreatic masses. Our aim is to highlight the diagnostic complexities of this condition and emphasize the need for a multidisciplinary approach to avoid unnecessary surgical interventions and ensure appropriate treatment.

## Introduction

IgG4-related disease is a rare, immune-mediated condition that can affect several organs, such as the pancreas, salivary glands, retroperitoneum, kidneys, lungs, and lymph nodes [1]. It is characterized by elevated serum IgG4 levels and IgG4-positive lymphocyte infiltration of the organs [1]. The main presenting symptoms include jaundice, abdominal pain, pruritus, steatorrhea, and new-onset diabetes mellitus [1]. IgG4-related autoimmune pancreatitis (AIP) is the most common manifestation of IgG4-related disease [1]. A challenge arises in differentiating IgG4-related AIP from disorders like pancreatic cancer due to their similarities [1,2]. It is important to differentiate between the two conditions to avoid unnecessary surgery and to provide appropriate treatment [1,2]. We report two cases of IgG4-related AIP presenting with pancreatic mass that were diagnosed and treated appropriately.

## Case presentation

Case 1

A 66-year-old male was referred with a history of jaundice, weight loss, and generalised body itching. He had no nausea, his stool was pale, and his urine was dark. His past medical history included rectal cancer in 2008 for which he underwent laparoscopic anterior resection. In addition to that, he had ischemic heart disease, type 2 diabetes, chronic kidney disease, and hypertension.

He had a previous episode of upper abdominal pain with an ultrasound showing gallstones. He had no history of foreign travel, no new sexual partners, and no history of recreational drug use. He very rarely took alcohol and had no family history of liver disease.

On examination, he had no symptoms of chronic liver disease, and his abdomen was normal. His investigations showed deranged liver enzymes, raised bilirubin, and elevated IgG4 level (4.6 g/L).

His abdominal ultrasound showed a hypoechoic solid mass near the pancreas measuring approximately 4.24 x 2.4 x 3.1 cm and the differential diagnosis included pancreatic mass or pathological lymph nodes.

**Figure 1:**
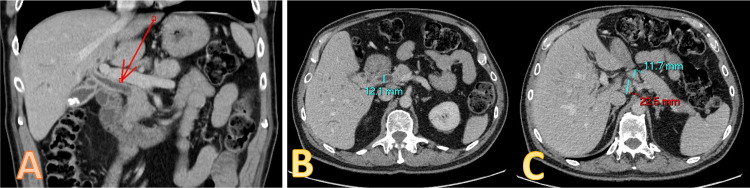
Abdominal contrast-enhanced computed tomography. (A and B) Diffuse common bile duct thickening and biliary dilatation. (C) Few peri-pancreatic slightly enlarged nodes.

A CT scan showed a pancreatic head mass, causing obstruction of the distal common bile duct (CBD) with diffuse CBD thickening and biliary dilatation. Portal, celiac, and para-aortic adenopathy were seen. There was no local recurrence of cancer at the rectal anastomotic site but enhancing lesions were seen bilaterally in the perinephric regions (Figure 1).

**Figure 2:**
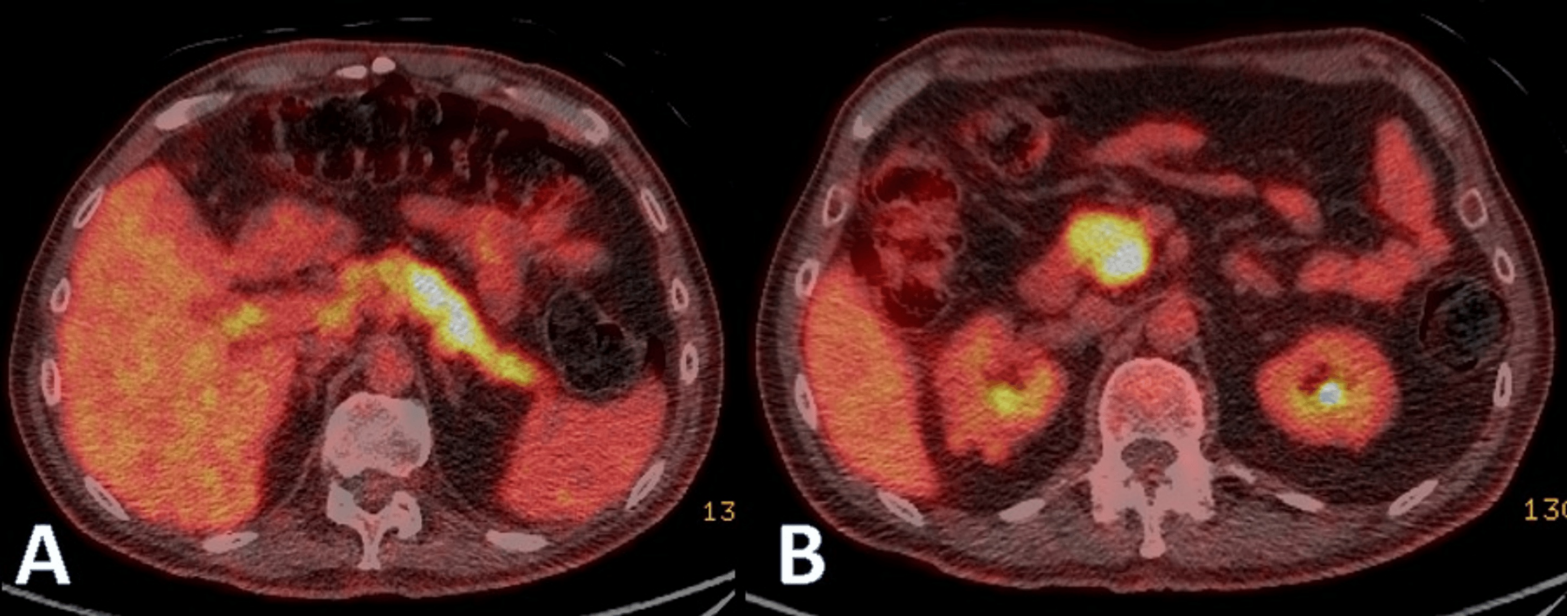
PET scan images. (A) Diffuse PET uptake throughout the pancreas. (B) Slightly more intense uptake in the head and uncinate process.

A PET scan showed diffuse uptake throughout the pancreas, slightly more intense in the head and uncinate process, and increased uptake in the nodular soft tissue surrounding both kidneys. Moderate uptake in relation to a circumferentially thickened CBD was noted. The metabolic appearances on PET raised the possibility of IgG4-related AIP, cholangiopathy, and renal disease (Figure 2).

**Figure 3:**
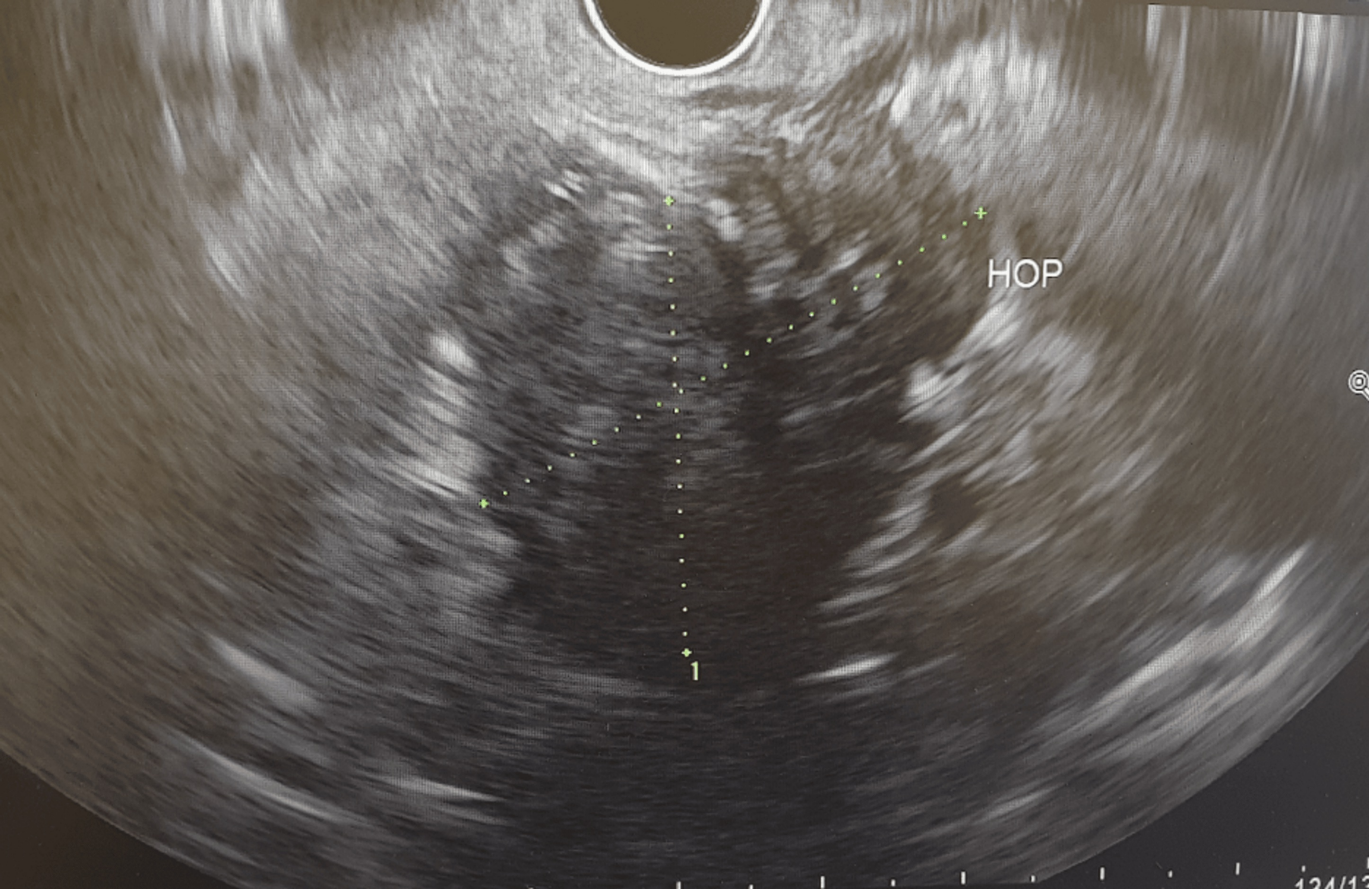
Endoscopic ultrasound showing pancreatic head mass.

An endoscopic ultrasound (EUS) scan was performed, and it showed thickened CBD walls with an irregular pancreatic head mass and a general hypoechogenicity of the rest of the pancreas. The pancreatic duct was not dilated and the features were in favour of IgG4-related AIP, but malignancy was possible (Figure 3).

EUS-guided biopsies from the pancreatic mass did not show clear malignant cells, so the case was discussed with the multidisciplinary team, and the decision was to treat the patient as IgG4-related AIP. Prednisolone 40 mg once per day was started and the liver function test showed significant improvement. However, diabetes got worse and the patient had a flare of his AIP on reducing steroids, hence azathioprine was added.

The follow-up investigations showed normalisation of his liver function tests and IgG4 levels (Table 1). His follow-up CT scan showed resolution of the pancreatic and perinephric lesions.

**Table 1:** Blood investigations before and after treatment.

Blood test	6/2022 (pre-steroid)	4/2024 (post-steroid)	Normal range
Albumin (g/L)	38	35	35-50 g/L
Total bilirubin	41	11	<21 µmol/L
Alanine transaminase (ALT)	231	22	7-56 units/L
Alkaline phosphatase (ALP)	431	89	44-147 units/L
Gamma-glutamyl transferase (GGT)	1035	44	9-48 units/L
IgG4	4.6	0.15	<1.35 g/L

Case 2

A 73-year-old female patient with chronic kidney disease, hypertension, and heart block on a cardiac pacemaker was referred in 2023 for deranged liver enzymes. She was asymptomatic and her physical examination was normal.

**Figure 4:**
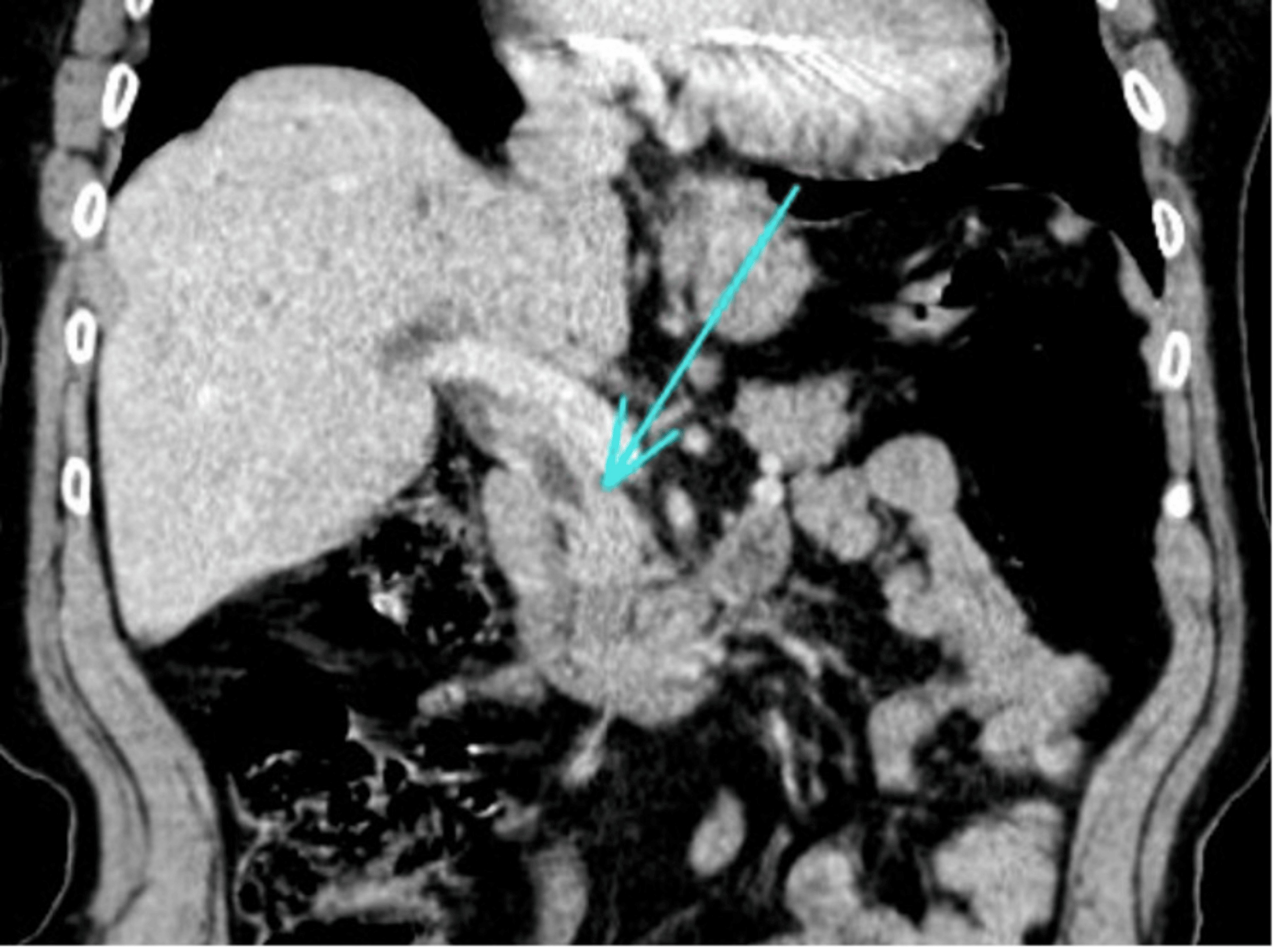
CT showing biliary dilatation with common bile duct thickening and abrupt transition and stricturing at the distal end.

Her abdominal ultrasound revealed biliary duct dilatation with no identifiable cause, and the CT scan showed moderate intrahepatic biliary duct dilatation with a markedly dilated CBD secondary to a mass within the head of the pancreas (Figure 4).

**Figure 5:**
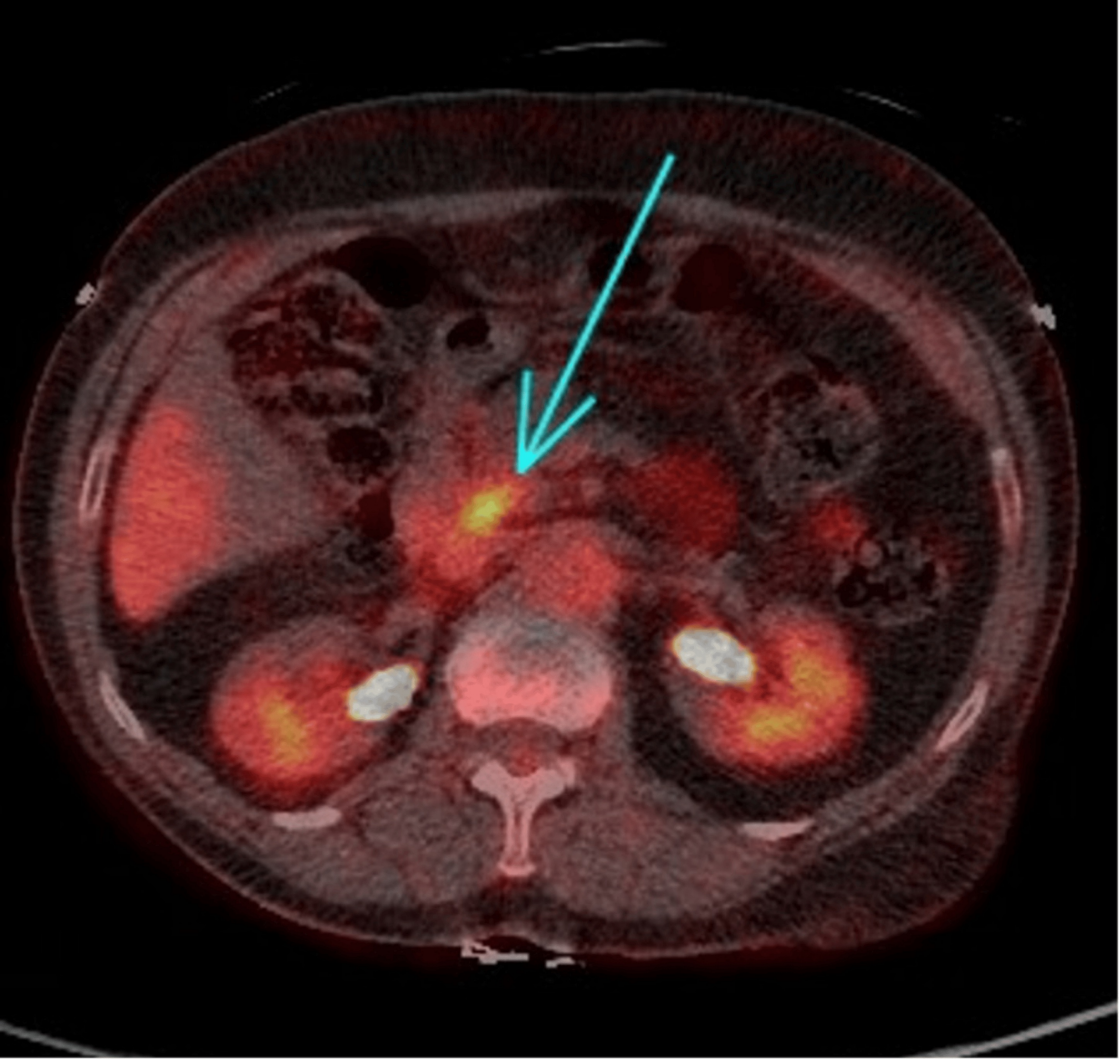
PET scan showing pancreatic head and distal common bile duct lesion.

**Figure 6:**
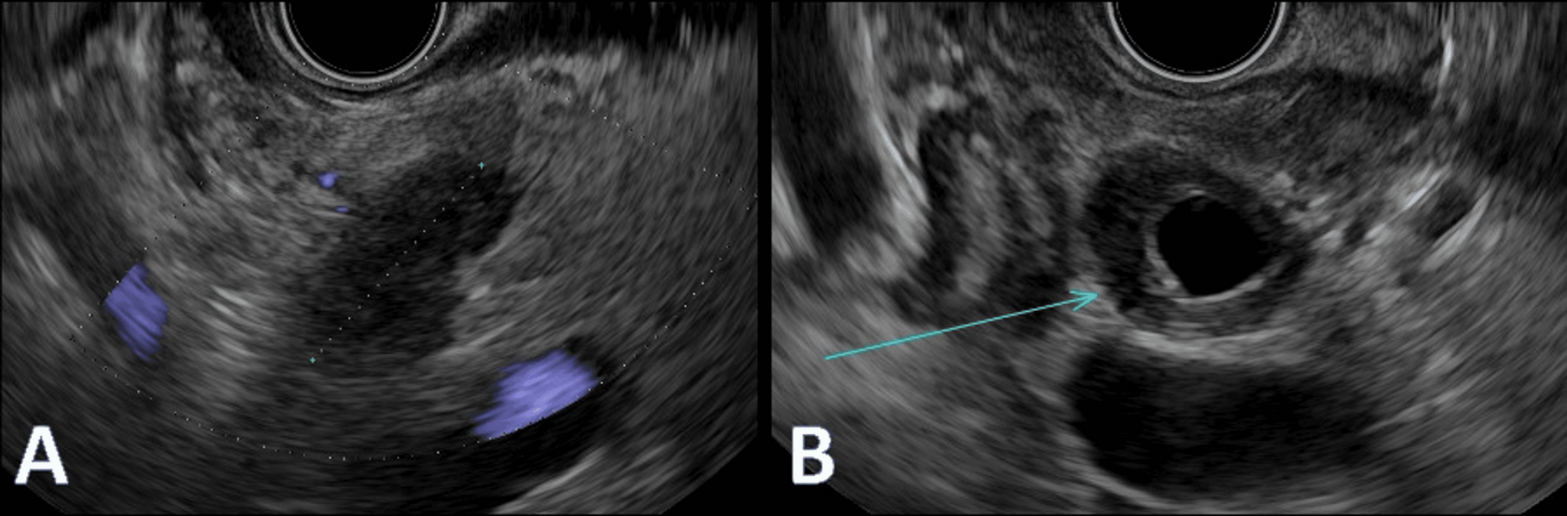
Endoscopic ultrasound. (A) A heterogeneous lesion at the pancreatic head. (B) Diffuse common bile duct thickening (blue arrow).

PET scan showed a pancreatic head lesion with no lymph node or distant metastases (Figure 5). EUS showed distal CBD concentric thickening (3 mm) with stricture and a small irregular mass suggestive of cholangiocarcinoma versus autoimmune cholangiopathy (Figure 6).

Endoscopic retrograde cholangiopancreatography (ERCP) was done and it showed a long CBD stricture. Cholangioscopy showed features suggestive of malignancy, hence, a CBD brushing was done for the stricture and a fully covered metallic stent was inserted.

The CBD cytology was non-diagnostic for malignancy but her raised IgG4 level (3.84 g/L) and histological findings of fibrosis and chronic inflammation raised the possibility of IgG4-related AIP. The patient was offered a choice between a Whipple's procedure or treatment for IgG4-related disease. She decided to take the treatment for IgG4 disease, so prednisolone was started.

Her follow-up blood investigations showed complete normalisation of her liver enzymes and IgG4 level (Table 2), and her CT showed resolution of the mass with steroid therapy, which was in favour of IgG4-related AIP.

**Table 2:** Blood investigations before and after treatment.

Blood test	2/2023 (pre-steroid)	4/2024 (post-steroid)	Normal range
Albumin (g/L)	35	39	35-50 g/L
Total bilirubin	21	7	<21* *µmol/L
Alanine transaminase (ALT)	535	11	7-56 units/L
Alkaline phosphatase (ALP)	411	84	44-147 units/L
Gamma-glutamyl transferase (GGT)	984	33	9-48 units/L
IgG4	3.84	0.96	<1.35 g/L

## Discussion

AIP comes in two forms: type 1 and type 2. Type 1 AIP is the type linked to IgG4-related disease (IgG4 RD) whereas type 2 AIP is commonly associated with inflammatory bowel disease [3]. IgG4-related AIP is a part of IgG4 RD, a condition that can affect various organs besides the pancreas, such as sclerosing cholangitis, salivary glands, and retroperitoneal tissues [3].

The average age of individuals with IgG4-related pancreatitis is 60 years and it tends to affect males more often [3,4]. Despite an increasing number of reported cases, the exact prevalence of this disease remains unknown.

Patients with IgG4-related AIP often present with abdominal pain, jaundice, weight loss, and continuous feeling of tiredness. The diagnosis of this condition includes clinical examination, imaging studies (CT and MRI), serum IgG4 levels, and histopathological examination of tissue samples [3].

Identifying IgG4-positive plasma cells in the pancreas is important to distinguish this disease from other pancreatic disorders. Although elevated serum IgG4 levels are commonly observed in cases of IgG4-related pancreatitis, they are not essential for making a diagnosis [3].

The similarity between AIP and pancreatic cancer presents diagnostic challenges, which result in delays in starting appropriate treatments [1]. This similarity highlights the significance of accurately distinguishing between the two conditions to prevent unnecessary surgeries due to clinical presentations alone [1]. EUS-guided needle aspiration and fluorine-18 fluorodeoxyglucose positron emission tomography (18F-FDG PET) are advised for differentiating between AIP and pancreatic cancer, along with elevated serum IgG4 levels [4]. 18F-FDG PET/CT is a useful tool in the diagnosis of IgG4 RD as it can reveal the multisystemic involvement of this illness [3,4].

Steroids are the first-line treatment for type 1 AIP. Imaging evaluations should be repeated two weeks after the therapy as a rapid response to a steroid is a typical feature of type 1 AIP, and an inadequate response can indicate an alternate diagnosis [3].

Around 15-60% of patients will experience relapse after weaning off the steroids. Immunomodulators such as azathioprine (AZA), methotrexate, and mycophenolate mofetil have been shown to be beneficial in the case of relapse or steroid resistance in type 1 AIP [3], in addition to the rituximab (RTX), which has been reported to be a successful approach for the treatment of type 1 AIP [3,4].

## Conclusions

In conclusion, the resemblance between IgG4-related AIP and pancreatic cancer can pose diagnostic challenges. A comprehensive diagnostic approach is needed and the dramatic response to glucocorticoid therapy further supports the diagnosis of IgG4-related AIP and highlights its value as both a diagnostic tool and treatment modality. It is important to consider the extrapancreatic manifestations of IgG4 disease. A thorough evaluation of other potential organ involvement can help in distinguishing IgG4-related AIP from pancreatic cancer. Distinguishing these conditions is important to provide better outcomes.
